# Spectrally non-overlapping background noise disturbs echolocation via acoustic masking in the CF-FM bat, *Hipposideros pratti*

**DOI:** 10.1093/conphys/coad017

**Published:** 2023-04-22

**Authors:** Jianwen Zou, Baoling Jin, Yuqin Ao, Yuqing Han, Baohua Huang, Yuyang Jia, Lijian Yang, Ya Jia, Qicai Chen, Ziying Fu

**Affiliations:** Hubei Key Laboratory of Genetic Regulation & Integrative Biology, School of Life Sciences, Central China Normal University, No.152 Luoyu Road, Wuhan City, Hubei Province, 430079, China; Hubei Key Laboratory of Genetic Regulation & Integrative Biology, School of Life Sciences, Central China Normal University, No.152 Luoyu Road, Wuhan City, Hubei Province, 430079, China; Hubei Key Laboratory of Genetic Regulation & Integrative Biology, School of Life Sciences, Central China Normal University, No.152 Luoyu Road, Wuhan City, Hubei Province, 430079, China; Hubei Key Laboratory of Genetic Regulation & Integrative Biology, School of Life Sciences, Central China Normal University, No.152 Luoyu Road, Wuhan City, Hubei Province, 430079, China; Hubei Key Laboratory of Genetic Regulation & Integrative Biology, School of Life Sciences, Central China Normal University, No.152 Luoyu Road, Wuhan City, Hubei Province, 430079, China; Hubei Key Laboratory of Genetic Regulation & Integrative Biology, School of Life Sciences, Central China Normal University, No.152 Luoyu Road, Wuhan City, Hubei Province, 430079, China; College of Physical Science and Technology, Central China Normal University, No.152 Luoyu Road, Wuhan City, Hubei Province, 430079, China; College of Physical Science and Technology, Central China Normal University, No.152 Luoyu Road, Wuhan City, Hubei Province, 430079, China; Hubei Key Laboratory of Genetic Regulation & Integrative Biology, School of Life Sciences, Central China Normal University, No.152 Luoyu Road, Wuhan City, Hubei Province, 430079, China; Hubei Key Laboratory of Genetic Regulation & Integrative Biology, School of Life Sciences, Central China Normal University, No.152 Luoyu Road, Wuhan City, Hubei Province, 430079, China

**Keywords:** spectrally non-overlapping noise, the Lombard effect, intensity tuning, *Hipposideros pratti*, echolocation signal, auditory sensitivity

## Abstract

The environment noise may disturb animal behavior and echolocation via three potential mechanisms: acoustic masking, reduced attention and noise avoidance. Compared with the mechanisms of reduced attention and noise avoidance, acoustic masking is thought to occur only when the signal and background noise overlap spectrally and temporally. In this study, we investigated the effects of spectrally non-overlapping noise on echolocation pulses and electrophysiological responses of a constant frequency–frequency modulation (CF-FM) bat, *Hipposideros pratti*. We found that *H. pratti* called at higher intensities while keeping the CFs of their echolocation pulses consistent. Electrophysiological tests indicated that the noise could decrease auditory sensitivity and sharp intensity tuning, suggesting that spectrally non-overlapping noise imparts an acoustic masking effect. Because anthropogenic noises are usually concentrated at low frequencies and are spectrally non-overlapping with the bat’s echolocation pulses, our results provide further evidence of negative consequences of anthropogenic noise. On this basis, we sound a warning against noise in the foraging habitats of echolocating bats.

## Teaser

*Hipposideros pratti* called at higher intensities while keeping the constant frequencies of their echolocation pulses consistent under spectrally non-overlapping background noise conditions. The noise could decrease auditory sensitivity and sharp intensity tuning, suggesting an acoustic masking effect. These results provide further evidence of negative consequences of anthropogenic noise.

## Introduction

Environmental noise arises from natural and ‘unnatural’ sources. Although most studies have shown that natural noise has negative consequences on animal behavior and echolocation (Brumm, 2013; Corcoran and Moss, 2017), such noise can also be beneficial. For example, animal sounds may indicate the presence of high-quality habitat for birds and frogs (Nocera *et al.,* 2006; Buxton *et al.,* 2015), female little torrent frogs may use cues from stream noise for sexual selection (Zhao *et al.,* 2017) and echolocating bats can use rainfall sounds to decide whether to initiate foraging trips (Geipel *et al.,* 2019). However, unnatural sources of noise, known as man-made or anthropogenic noise, is nearly unanimously considered to have negative impacts (Gomes *et al.,* 2021). Both natural and anthropogenic noise can impede animal foraging to a similar extent due to acoustic masking, reduced attention, and/or noise avoidance (Luo *et al.,* 2015). Compared with reduced attention and noise avoidance, acoustic masking has been thought to occur only when the signal and background noise spectrally and temporally overlap.

Nearly all echolocating bats emit ultrasonic sounds and capture echoes to construct critical information about their environment. Because the energy of anthropogenic noise sources is usually concentrated at lower frequencies (Warren *et al.,* 2006), it was believed that anthropogenic noise does not mask such echolocation signals. However, even if echolocating bats use ultrasounds, their auditory system still responds to low-frequency sounds. For example, although the echolocation calls of *Hipposideros pratti*, a constant frequency–frequency modulation (CF-FM) bat, has a dominant resting frequency (CF2) of ~60 kHz (Fu *et al.,* 2019), there are still some inferior collicular neurons with a best frequency (BF) of ~20 kHz (Cui *et al.,* 2021). Furthermore, echolocating bats also communicate with conspecifics using low-frequency social calls (Smotherman *et al.,* 2016; Håkansson *et al.,* 2022). Although the most sensitive responses occur in response to the BF, the auditory neurons also respond to a range of frequencies, which is described by frequency tuning curve. For instance, in our previous study, we found that the BF of an inferior collicular neuron is ~38 kHz. As such, this neuron is most sensitive to sound at 38 kHz, but it also responds to frequencies ranging from 10 to 50 kHz (Fig. 5A in Cui *et al.,* 2021). Although some neurons have relatively narrow frequency tuning, most neurons have broad tuning curves (Casseday and Covey, 1992), especially neurons in the auditory periphery and lower level of the central auditory system (Suga, 1995). Therefore, if the noise frequency spectrum overlaps with the frequency tuning range of a neuron, the noise may affect the neuronal responses, even without overlapping the BF. Moreover, there are complex connections among neurons of different BFs (Kato *et al.,* 2017). Thus, at least in the central auditory system, the effect of noise on neurons of certain BFs may influence other neurons that possess different BFs. Taken together, it is reasonable to suppose that echolocation signals may be masked by spectrally non-overlapping noise.

In this study, we tested the hypothesis that spectrally non-overlapping noise can disturb bat echolocation via acoustic masking. We used the CF-FM bat, *H. pratti*, in the present study. The echolocation signals of the bat usually consist of 3 harmonics (H1 – H3), and each harmonic includes a rather long CF component (CF1 – CF3) and an invariably terminated brief FM component (FM1 – FM3) (Fu *et al.,* 2019). The CF1 is ~30 kHz, and the FM1 sweeps downward ~4.5 kHz from the CF1 (Fu *et al.,* 2019). As such, the spectrally non-overlapping noise used in this study was white noise in the 1 to 20 kHz frequency range. We first recorded the echolocation signals of the bat under control conditions (silence) and 60 dB sound pressure level (SPL) background noise. We then tested the effects of the noise on auditory sensitivity. We predicted that the spectrally non-overlapping noise would change the echolocation signal and decrease auditory sensitivity, suggesting a disturbance via acoustic masking.

## Materials and Methods

### Ethics approval

All experiments were conducted with the approval of the Institutional Animal Care and Use Committee of Central China Normal University, Wuhan, Hubei, People’s Republic of China (Permit Number: CCNU-IACUC-2022-011). The electrophysiological recordings were performed under sodium pentobarbital anesthesia, and all efforts were made to minimize animal suffering.

### Animals and housing

Seven *H. pratti* (three males and four females; 60.5 ± 14.7 (43.5 – 85.4) g body weight) were used in the present study. Animals were wild-caught in a cave (N: 29°26′0.32″; E: 114°01′20.49″) near Xianning City of Hubei Province, China. Three bats (1 male and 2 females) were used in the echolocation behavior study. The remaining 4 bats were used in the electrophysiological study. Bats were socially housed in an animal room (dimensions: 3.0 m × 3.0 m × 3.0 m) at the Central China Normal University, Wuhan, China. All bats were exposed to the local photoperiod with a regulated air temperature (24 ± 2°C) and humidity (>60% relative humidity). The bats had *ad libitum* access to water and food (mealworms). Bats were examined daily for any sign of weakness, including an empty stomach or slow responses to being handheld, and bats observed to be in poor physiological condition were excluded from that day’s experiment.

### Recordings of echolocation pulses

The bats were acclimated to the laboratory conditions for several days before recordings of echolocation pulses. And then, we trained the bat to hang stably on the ceiling of the experimental anechoic cage. During each recording, the recording microphone was placed 1 m below the bat in the bat’s frontal azimuth space. The microphone membrane was pointed toward the bat’s nose as best as possible. The echolocation pulses were recorded from each bat in isolation using an ultrasound detector (Petterson D1000X; Pettersson Elektronik AB, Uppsala, Sweden) at a sampling rate of 384 kHz. Before each recording, we waited several minutes until the bat got used to captivity. Each bat was recorded one to three times for 20 s, with a 5-s silence control, 10 s with 60-dB SPL white noise and 5-s silence after the noise. The gain control was adjusted to avoid saturation of the recording system. The gain was unchanged in the same recording, and the microphone calibration was carefully done for each gain used.

The background white noise (1 – 20 kHz) was generated digitally using Tucker-Davis Technologies (TDT, Alachua, FL) system III hardware and OpenEX software. The noise was presented through an electrostatic speaker (ED1, TDT) under acoustic free-field conditions. White noise was generated continuously and attenuated (PA5, TDT) to an intensity of 60 dB SPL in the frequency range of 1 to 20 kHz around the bat’s pinna. The noise intensity was calibrated with a one-fourth–inch microphone (4939, B&K, Narum, Denmark). The output of the loudspeaker was expressed in decibel SPL in reference to the 20-μPa root mean square.

### Pulse analysis

**Figure 1:**
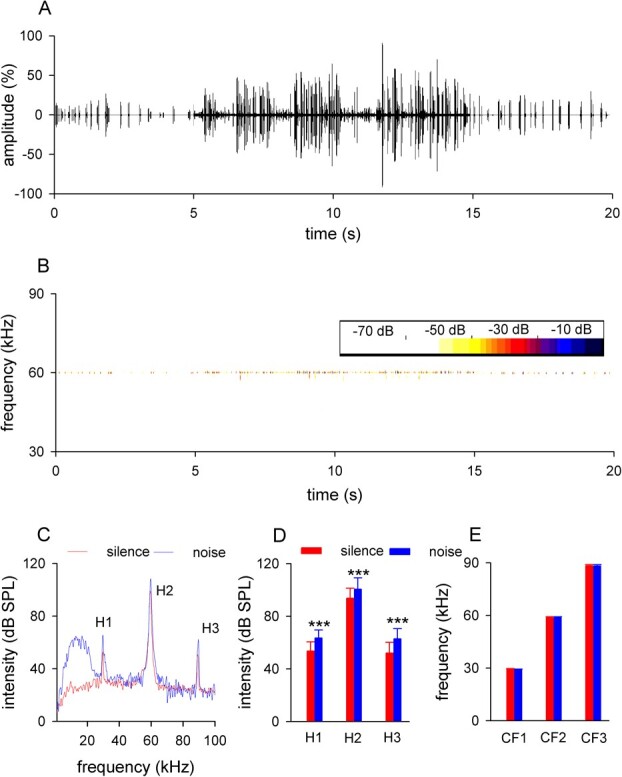
The effects of spectrally non-overlapping noise on echolocation pulses of *H. pratti*. A–B, a representative echolocation call trail of a bat before (0 – 5 s), during (5 – 15 s) and after (15 – 20 s) the spectrally non-overlapping background noise showed an increase in the call rate and intensity during the noise (A) and the nearly constant CF2 (B). The thicker center line between 5 and 15 s in (A) was due to the noise. C, a representative power spectrum of echolocation pulses under silent and noisy conditions. D, Comparison of the mean intensities of each harmonic for all bats between silent and noisy conditions, showing that *H. pratti* increased the intensities of H1 – H3 during the noise. E, Comparison of the mean CFs of each harmonic for all bats between silent and noisy conditions, showing that *H. pratti* kept the CF1 – CF3 unchanged during the noise. ^***^, *p* < 0.001.

The echolocation pulses were analyzed with the BatSound pro 3.31b (Pettersson Elektronik AB, Uppsala, Sweden), with a fast Fourier transformation (FFT) size of 8192 points and a Hanning window with a cursor and visual determination on a screen. The intensities and frequencies of echolocation pulses were collected from the power spectrum (Fig. 1C).

### Acoustic stimulation and auditory brainstem response (ABR) recordings

For acoustic stimulation, continuous sine waves from a function generator (33500B, Agilent, Santa Clara, CA) were formed into pure tone pulses or bursts (10 ms with a rise-decay time of 0.5 ms, delivered at 5 pulses/s, hereafter identified as CF sound) by a custom-made tone burst generator driven by a stimulator (Master 8, AMPI, Jerusalem, Israel). The sounds were amplified after passing through a decade attenuator (LAT45, Leader, Kohokuku, Japan) before being fed into a small loudspeaker (AKG model CK 50, 1.5 cm in diameter, 1.2 g, frequency response 1 – 100 kHz). The loudspeaker was placed 20 cm away from the animal’s ear and 30 degrees contralateral to the recording site. Calibration of the loudspeaker was conducted using a one-fourth–inch microphone (4939, B&K, Denmark) placed at the animal’s ear using a measuring amplifier (2610, B&K, Denmark). The output of the loudspeaker was also expressed in decibel SPL in reference to the 20-μPa root mean square. A frequency-response curve of the loudspeaker was plotted to determine the maximal available sound intensity at each frequency. The maximal stimulus intensity ranged from 110 to 125 dB SPL between 10 and 80 kHz but declined to 80 dB SPL almost linearly from 80 to 100 kHz.

ABR recordings were captured in a custom-made double-wall soundproof room (temperature 28 – 30°C). The ceiling and inside walls of the room were covered with 8-cm convoluted polyurethane foam to reduce echoes. The bat was administered a mixture of Nembutal (40 mg/kg) and xylazine (5 mg/kg) and it was then placed inside a bat holder. Additional doses of the anesthetic mixture were administered during later phases of recording if the bats showed any signs of discomfort. Three needle-electrodes (NS-S83018-R9–10, NY) were placed subcutaneously on the bat’s head. The recording electrode was inserted at the caudal midline of the head, close to the brainstem. The reference electrode was inserted at the dorsal midline of the head between the ears. The ground electrode was inserted directly at the base of the right ear. At the end of each recording, the electrodes were manually removed and cleaned with 75% ethanol. There was no evidence of infection at the sites of needle insertion.

**Figure 2:**
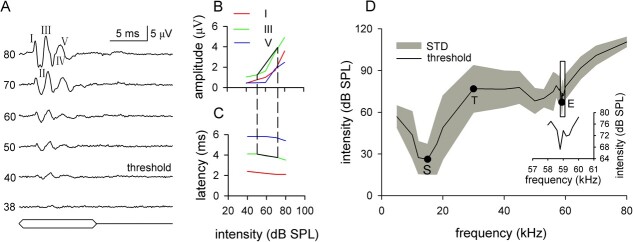
ABR audiogram of *H. pratti*. A, representative ABR waveforms to 10 ms, 20 kHz pure tones of increasing stimulus intensity. The sound stimulation is sketched below the ABR waveform. B–C, the ABR amplitude- and latency-intensity functions of the representative ABR in (A). The black solid and broken lines showed the method for calculating slope_a_ and slope_l_. D, the mean audiogram of *H. pratti*. Inset, magnified view of boxed region in (D).

The ABR signal was amplified (10 000×) and filtered (0.3 – 3.0 kHz) using a biological electrical signal amplifier (ISO-80, WPI, Sarasota, FL), digitized with an analog-to-digital converter equipped with a data acquisition system (Digidata 1440A, Axon, Sunnyvale, CA; sampling rates, 100 kHz) before being stored in a computer database (Kaitian 4600, Lenovo, China). The signal also was monitored with an oscilloscope (DSO-X 2014A, Agilent). All stimuli were repeated 256 times at a repetition rate of 5 Hz to collect the averaged ABRs using PCLAMP 8.1 software (Axon Instruments). The ABR waveform typically consisted of five peaks (I, II, III, IV and V), with three dominants (I, III and V) (Fig. 2A). The amplitude of each ABR waveform was defined as the peak value of the waveform. The latency of each ABR waveform was measured from the time of stimulus onset to the time of the waveform peak.

### ABR threshold estimation

The sound frequencies used to obtain ABRs ranged from 5 to 80 kHz. At each sound frequency, the sound intensity varied from 0 dB SPL to the maximal available sound intensity at each frequency to obtain the ABR threshold. Keeping the sound frequency constant, the sound intensity was increased in 10-dB steps until a distinct ABR waveform was observed. Then, the sound intensity was decreased in 2-dB steps until the ABR waveform was no longer visible. The lowest sound intensity that evoked a visible ABR was deemed to be the ABR threshold at that frequency. The ABR thresholds were established at a series of frequencies to generate the audiogram for each bat. The frequencies started at 5 kHz and increased to 55 kHz with a step size of 3 to 5 kHz; from 55 to 62 kHz, the step size was 0.2 to 0.5 kHz and reverted to 3 to 5 kHz for frequencies at 62 to 80 kHz. We used a small step size for 55 to 62 kHz because the dominant frequency was ~60 kHz, and we expected that the thresholds would change drastically in this region.

### ABR amplitude- and latency-intensity functions

The ability of *H. pratti* to process sound intensity was quantitively studied by the slope of ABR amplitude- and latency-intensity functions, which described the relationship between amplitudes and latencies of three dominant waves and the sound intensities. The sound intensities started at the threshold and was increased with 2- to 10-dB steps. To obtain the slope, we first established the dynamic range, which was defined as the intensity range from 20% below the maximal to 20% above the minimal amplitude of each ABR wave. The slope of an amplitude-intensity function (slope_a_) was obtained by dividing the amplitude change of each ABR wave within the dynamic range by the dynamic range (Fig. 2B). Similarly, the slope of a latency-intensity function (slope_l_) was obtained by dividing the latency change of each ABR wave within the dynamic range by the dynamic range (Fig. 2C). Because the wave amplitude normally increased with increasing sound intensity, slope_a_ was positive. Conversely, slope_l_ was negative because wave latency usually decreased with increasing sound intensity. Therefore, a higher slope_a_ and lower slope_l_ reflect sharper intensity tuning.

### Data analysis

Data were processed and plotted using Sigmaplot, version 10.0 (Systat Software, San Jose, CA). The results are expressed as means ± standard deviations (mean ± SD) and then analyzed using SPSS, version 13.0 (SPSS Inc., Chicago, IL). In the experiment studying the effects of noise on bat echolocation signals, a total of 741 high-quality pulses were recorded (135 in silent and 606 in noisy conditions). The differences in pulse intensities and dominant frequencies between silent and noisy conditions were tested using the Mann–Whitney *U* test. In the experiment studying the effects of noise effect on bat electrophysiological responses, paired *t* tests were used to test the differences in noise effects on intensity tuning. In all tests, *p* < 0.05 was considered to be statistically significant.

## Results

### The Lombard effect induced by spectrally non-overlapping background noise

Echolocation signals were recorded before, during and after spectrally non-overlapping noise (1 – 20 kHz) in three *H. pratti* (1 male, 2 females) bats. We noted that bats were more active in echolocation calls during the noisy condition compared with the silent control condition (Fig. 1A). On average, call rates were 4.5 Hz during the silent control and increased to 20.2 Hz during the noisy condition.

Excepting calling at high rates*, H. pratti* also called at high intensities under the noisy condition (Fig. 1A). The power spectrum of representative echolocation signals showed that the bat increased the intensities of all harmonics during the noisy condition (Fig. 1C). We found that the intensity of H1 – H3 increased significantly during the noisy condition (Fig. 1D; H1, 53.6 ± 7.0 vs 63.4 ± 6.1 dB SPL, *p* < 0.001; H2, 93.6 ± 7.7 vs 100.6 ± 8.6 dB SPL, *p* < 0.001 and H3, 52.0 ± 8.2 vs 62.8 ± 7.7 dB SPL, *p* < 0.001), indicating that the spectrally non-overlapping noise also can induce the Lombard effect. However, the spectrogram of the representative echolocation signals showed that the bat kept the CF components of its echolocation signals nearly unchanged during the noisy condition (Fig. 1B), and the statistical analysis indicated that the CF component of each harmonic (CF1 – CF3) was kept consistent under the noisy condition (Fig. 1E; H1, 29.4 ± 0.3 vs 29.0 ± 0.4 kHz, *p* > 0.05; H2, 58.8 ± 0.5 vs 58.7 ± 0.5 kHz, *p* > 0.05 and H3, 88.2 ± 0.8 vs 88.2 ± 0.7 kHz, *p* > 0.05). In summary, *H. pratti* adjusted their echolocation signals under the spectrally non-overlapping noisy conditions by calling at higher rates and intensities while keeping the CF component consistent. This suggests that spectrally non-overlapping noise can potentially disturb the echolocation ability of *H. pratti*.

### ABR audiogram of *H. pratti*

To test the hypothesis that spectrally non-overlapping noise can disturb echolocation in *H. pratti* via acoustic masking, we studied the effects of the noise on auditory sensitivity using ABR recordings, which are acquired rapidly and non-invasively. The ABRs of *H. pratti* were quite uniform and stable across all bats, and the waveforms were easily detected (Fig. 2A). When stimulated with high-intensity sounds, the ABR waveform consisted of five peaks (wave I, II, III, IV and V) with three dominant waves (wave I, III, and V), closely resembling the ABRs recorded from *Carollia perspicillata* (Wetekam *et al.,* 2020; Lattenkamp *et al.,* 2021). The three dominant waves were readily detected at relatively low intensities, whereas waves II and IV were fused with wave III and wave V, respectively. Therefore, we only quantitatively analyzed the three dominant waves.

The ABR wave amplitude increased and its latency decreased with the increasing sound intensities (Figs 2A–C); the threshold was defined as the lowest sound intensity that evoked a visible ABR (40 dB SPL in Fig. 2A; see Methods for detail). An ABR audiogram of each bat was generated by establishing ABR thresholds at a series of frequencies. An overall audiogram of *H. pratti* was generated by averaging the audiograms of 4 bats (Fig. 2D). It was obvious that there were two sensitive areas in the ABR audiogram; one at ~15 kHz, which was thought to be related to social communication (social frequency area) and one at ~59 kHz, which was thought to be related to echolocation (echolocation frequency area), and the thresholds of the high-frequency area were relatively high (Fig. 2D). A magnified view of the boxed region showed that the threshold changed ~10 dB in the 58- to 60-kHz range in the echolocation frequency area (Fig. 2D inset). The audiogram showed an insensitive peak between two sensitive areas at ~30 kHz, which was in the frequency range of CF1 (Fig. 2D).

### Effects of spectrally non-overlapping noise on the ABR threshold

Based on the audiogram of *H. pratti*, we chose three representative frequencies to study the effects of spectrally non-overlapping noise on the ABR threshold (dots in Fig. 2D), i.e. the most sensitive frequency in the social frequency area (hereafter referred to as “S” for convenience), the most sensitive frequency in the echolocation area (hereafter referred to as “E” for convenience) and 30 kHz, which is ~CF1 (hereafter referred to as “T” for convenience), respectively. Previous study reported that the psychoacoustic threshold of the FM bat *Eptesicus fuscus* shows a day-to-day variability of up to 6 dB (Simmons *et al.,* 2016). Because the protocols testing the effects of spectrally non-overlapping noise on the ABR threshold took ~1 h, we first checked the pre-noise ABR threshold variability over 1 h.

**Figure 3:**
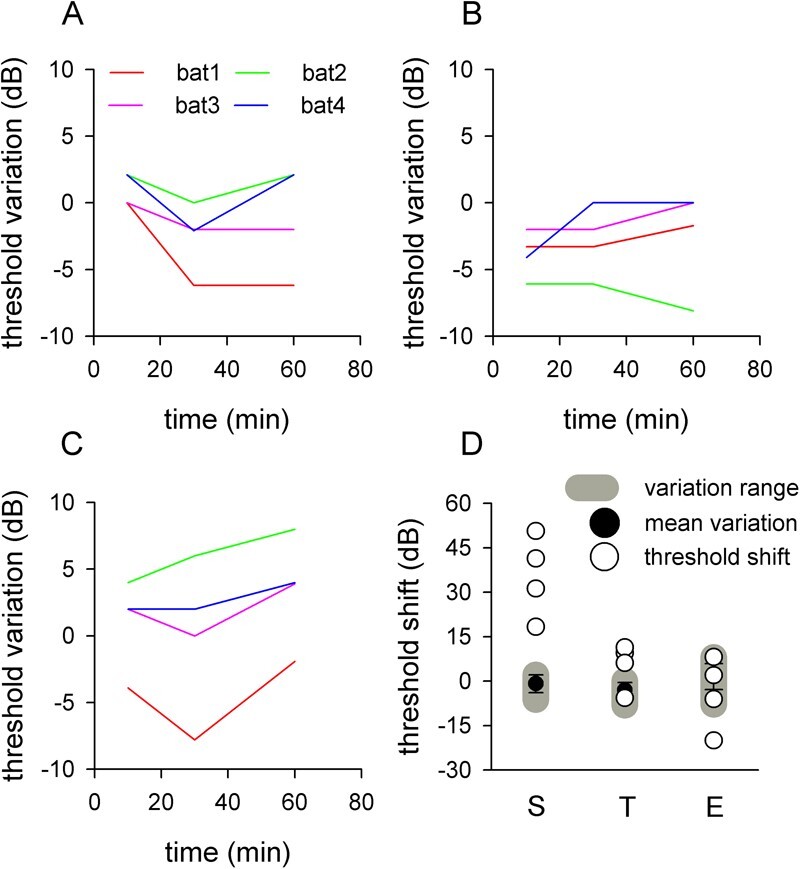
The effects of spectrally non-overlapping noise on the auditory sensitivity of *H. pratti*. A–C, ABR threshold variation in an hour under silent condition when stimulated with frequency S (A), T (B) and E (C). D, ABR threshold shift during the noise condition.

The ABR thresholds at frequencies S, T and E were first obtained as controls and then established again at 10 min, 30 min and 1 h after the control to test the pre-noise ABR threshold variations (Figs 3A–C). The ABR threshold variations for all 4 bats were −6 to 2 dB when stimulated with frequency S (Fig. 3A), −8 to 0 dB when stimulated with frequency T (Fig. 3B) and −7 to 8 dB when stimulated with frequency E (Fig. 3C). These data indicate that an ABR threshold shift of 8 dB is likely a normal variation in *H. pratti*.

The effects of spectrally non-overlapping noise on the ABR threshold were studied by observing threshold variation after applying the noise. At frequency S, the threshold increased from 18 to 41 dB in noisy condition; this dramatically exceeded the normal range of variation (Fig. 3D left). At frequency T, although the threshold increased beyond the normal variation in 3 bats (6 – 11 dB), one bat had a threshold shift (−5 dB) within the normal variation range (Fig. 3D middle). In contrast to frequency S and T, frequency E showed a threshold shift within the normal variation range in 3 bats (−6 – 8 dB); the remaining bat showed a markedly lower threshold in noise (−20 dB shift) when stimulated with frequency E (Fig. 3D right). We reexamined the threshold shift of the latter bat on a subsequent day and obtained similar results. Together, the spectrally non-overlapping noise dramatically increased the ABR threshold of sounds with frequencies within the bat’s social frequency range, slightly increased the ABR threshold of CF1 sounds in most bats (3/4) and had almost no effect on the ABR threshold of sounds with frequencies in the echolocation frequency range of most bats (3/4), which might be the possible reason why the bats called at higher intensities under the noise condition.

### Effects of the background noise on intensity tuning

Our data showed that *H. pratti* called louder and had a higher ABR threshold under spectrally non-overlapping noise conditions, suggesting that an acoustic masking effect was involved. A large number of studies have indicated that masking noise can sharpen the intensity tuning of single auditory neurons (Palmer and Evans, 1982; Phillips and Hall, 1986; Cui *et al.,* 2021). However, the effects of background noise on the intensity tuning of ABR have not been extensively investigated in echolocating bats. Here, we examined both the slope_a_ and slope_l_ of three dominant waves of ABR waveforms under silent control and noisy conditions. Because the noise was most effective on the ABR threshold in frequency S, we studied the noise effects on intensity tuning using the same frequency.

**Figure 4:**
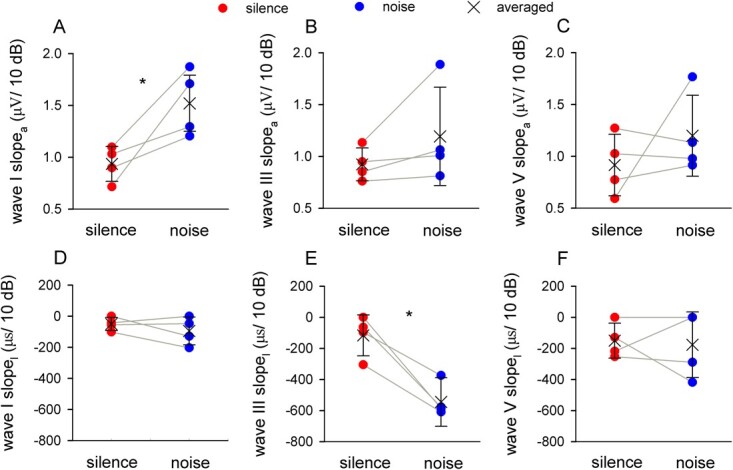
The effects of spectrally non-overlapping noise on intensity tuning. A–C, effects of the noise on slope_a_ of wave I (A), wave III (B), and wave V (C). D–F, the effects of the noise on slope_l_ of wave I (D), wave III (E), and wave V (F). *, *p* < 0.05.

*H. pratti* had a higher averaged slope_a_ for wave I, III and V (Fig. 4A–C) under the noisy condition, and wave I showed a significant increase (0.93 ± 0.16 vs 1.52 ± 0.27 μV/10 dB, *p* < 0.05; Fig. 4A). Accordingly, *H. pratti* had a lower averaged slope_l_ for all three dominant waves under the noisy condition (Figs 4D–F), and wave III was significant (−116.4 ± 132.0 vs −544.5 ± 155.8 μs/10 dB, *p* < 0.05; Fig. 4E). These data suggest that the noise can sharpen the intensity tuning of ABR. Taken together, the noise induced higher auditory thresholds and more sharp intensity tunings of ABRs, indicating acoustic masking effects of the noise.

## Discussion

*H. pratti* emits CF-FM signals for echolocation. The CF1 is ~30 kHz, and FM1 sweeps downward ~4.5 kHz from CF1. As such, we used 60-dB SPL, 1- to 20-kHz white noise to study the effects of spectrally non-overlapping noise on echolocation signals and ABRs of these bats. Our data showed that, during noisy conditions, *H. pratti* increase H1 – H3 intensities while keeping CF1 – CF3 constant. ABR recordings showed that *H. pratti* have higher ABR thresholds when they are stimulated with sounds at frequencies ~CF1 or lower under the noise condition due to acoustic masking. In addition, the noise can sharpen the ABR intensity tuning by increasing slope_a_ or decreasing slope_l_, further supporting the acoustic masking effects of the noise. Taken together, these results indicate that the spectrally non-overlapping noise can potentially disturb echolocation via acoustic masking.

### The Lombard effect

Many animals increase their vocal intensity when communicating in noisy environments using auditory system; this well-known phenomenon is called the Lombard effect, which was first described in humans >100 years ago (Brumm and Zollinger, 2011). The Lombard effect is thought to maintain an optimal signal-to-noise ratio (SNR) under noisy conditions (Luo *et al.,* 2018) and is commonly used by vertebrates (Kunc *et al.,* 2022). In this study, we showed that spectrally non-overlapping noise can also induce the Lombard effect (Figs 1A–C). These findings are consistent with a recent laboratory study (Lu *et al.,* 2020). Therefore, we conclude that low SNR is not the only trigger for the Lombard effect, or the bats are still trying to maintain a perceptual SNR does not depend only on the carrier frequencies of the noise. Multiple field studies have shown that echolocating behavior is disturbed by anthropogenic noise, which is usually spectrally non-overlapping (Siemers and Schaub, 2011; Bunkley *et al.,* 2015; Bunkley and Barber, 2015; Luo *et al.,* 2015; Wang *et al.,* 2022). However, whether echolocating bats increase the intensity of their calls in response to spectrally non-overlapping noise under field conditions still needs to be verified.

In addition to the Lombard effect, some animals including echolocating bats also adjust their call frequency to avoid noise disturbance (Tressler and Smotherman, 2009; Hage *et al.,* 2013). However, two species of *Hipposideros* bat (*Hipposideros armiger* and *H. pratti*) have been shown to maintain relatively stable dominant frequencies under spectrally overlapping noise conditions (Lu *et al.,* 2020; Zhang *et al.,* 2021). In this study, we found that *H. pratti* maintained the CF1 – CF3 of their echolocation signals constant during the spectrally non-overlapping conditions (Fig. 1E). Compared with the CF-FM bat *Rhinolophus ferrumequinum*, which adjust their dominant frequency under some noisy conditions, *Hipposideros* bats are short–CF-FM bats (Zhang *et al.,* 2021). Long–CF-FM bats have extremely sharp frequency tuning and stable dominant frequency adapting for precise Doppler-shift compensation (Zhang *et al.,* 2019); the dominant frequencies in short–CF-FM bats are less stable (Schoeppler *et al.,* 2018). Thus, we speculate that sharp frequency tuning and stable call frequency can help long–CF-FM bats to obtain higher SNR by adjusting the call frequency. Additional data on more CF-FM bats are needed to better understand these differences between species.

### Audiogram of CF-FM bats

Behavioral and electrophysiological studies have shown that the audiogram of long–CF-FM bats has a sharp and high sensitivity area around the dominant frequency of their echolocation signals (Neuweiler *et al.,* 1971; Long and Schnitzler, 1975). For example, the behavioral audiogram of *R. ferrumequinum* can change nearly 40 dB in the 2-kHz region around its dominant frequency (Long and Schnitzler, 1975). Unlike long–CF-FM bats, the electrophysiological audiogram of *H. pratti*, a short–CF-FM bat, showed only a ~10-dB change around its dominant frequency (Fig. 2D inset). It is recognized that long–CF-FM bats have a disproportionately high representation of sharply tuned neurons in the range of their dominant frequencies, whereas the sharpness of auditory neurons in short–CF-FM bats is relatively low (Schnitzler and Denzinger, 2011). Because the audiogram is delineated by the distribution of auditory neurons of the lowest thresholds, we considered that the different neural response properties between the long– and short–CF-FM bats were responsible for the difference in their audiograms.

It is interesting to note that the insensitive area of the audiogram peaked at frequencies around the CF1 (Fig. 2D). Because the H1 of echolocation signals was ~40 dB less intense than the H2 (Fig. 1D), the less insensitive of this frequency range made the H1 unlikely to be heard except by the calling bat. Therefore, the combination-sensitive neurons, which was first described in **Pteronotus* parnellii* and possibly exist in the *H. pratti* (Mora *et al.,* 2013), can only be activated by the bat’s own call; this is a high-efficiency mechanism to overcome jamming signals from other bats (Ulanovsky and Moss, 2008).

Similar to most ABR audiograms recorded from both FM and CF-FM bats (Wenstrup, 1984; Wetekam *et al.,* 2020; Lattenkamp *et al.,* 2021), the audiogram of *H. pratti* had high sensitivity at low frequencies and relatively low sensitivity at high frequencies (Fig. 2D). Single-cell recordings showed that many low- and high-frequency neurons respond to low-frequency sounds (Casseday and Covey, 1992; Suga, 1995; Cui *et al.,* 2021), which might account for the high sensitivity at the low-frequency area of the audiogram.

### Spectrally non-overlapping noise

Environmental noise can impede animal foraging via three main potential mechanisms, which are acoustic masking, reduced attention, and noise avoidance (Luo *et al.,* 2015). Compared with reduced attention and noise avoidance, acoustic masking was thought to occur only when the signal and background noise are spectrally and temporally overlapping. However, we showed that spectrally non-overlapping noise can potentially disturb echolocation of *H. pratti* via acoustic masking (Fig. 3, 4). We believe that spectrally non-overlapping noise can mask the echolocation signal of *H. pratti* through two potential mechanisms.

Firstly, spectrally non-overlapping noise can mask the auditory responses of neurons in the frequency range of H1. Although neurons in the frequency range of the bat’s dominant frequency are sharply tuned, neurons in the frequency range of H1 respond to a wide range of frequencies (Cui *et al.,* 2021). Thus, spectrally non-overlapping noise can overlay parts of the responsive range of these neurons and thereby mask their responses. Because foraging behaviors are largely dependent on the functions of combination-sensitive neurons (Ulanovsky and Moss, 2008), which are activated by H1, the auditory masking of spectrally non-overlapping noise on neurons in the frequency range of H1 will disturb foraging behaviors.

Secondly, spectrally non-overlapping noise can mask the auditory responses of neurons in the frequency range of echolocation signals through local neural circuits. The response properties of each neuron are shaped both directly (through the synapse on the examined neuron) and indirectly (by connecting neurons in local neural circuits). The audiogram of *H. pratti* showed that their auditory systems responded to a frequency range of 5 to 80 kHz. Therefore, spectrally non-overlapping noise can activate some neurons, and these neurons may synapse on neurons in the frequency range of echolocation signals through local neural circuits, activating an inhibitory neural network (Seybold *et al.,* 2015; López-Jury *et al.,* 2021; Wetekam *et al.,* 2022).

### Possible biological relevance of this study

Our present study shows that 60-dB SPL, 1- to 20-kHz white noise can potentially disturb the echolocation behavior of *H. pratti* via acoustic masking. The noise used in this study was in the frequency range of the bat’s communication calls and some anthropogenic noises, such as airport noise (Wang *et al.,* 2022) and traffic noise (Geipel *et al.,* 2019). Although the bats are used to and resilient to their communication calls, the constant anthropogenic noise could have stronger effects on the biosonar system of the bats. For example, echolocating bats may call at higher intensities when exposed to anthropogenic noise conditions; these efforts will increase energy use and help attract predators (Gomes *et al.,* 2021). In addition, the noise may also decrease the auditory sensitivity, initiating an acoustic masking effect. Our results further demonstrated the negative consequences of anthropogenic noise, and we call for noise management in foraging habitats of echolocating bats.

## Funding

This work was supported by grants from the National Natural Science Foundation of China (32270534) and the Fundamental Research Funds for the Central Universities (CCNU22JC009).

## Author contributions

The study was conducted in the laboratory at Central China Normal University. Z.F. contributed to the conception and design of the study, acquisition, analysis and interpretation of the data and drafting the manuscript. J.Z., B. J., Y. A., Y. H., B. H. and Y. J. contributed to acquisition, analysis and interpretation of the data and revising the draft critically for important intellectual content. L.Y., Y.J. and Q.C. contributed to revising the draft critically for important intellectual content. All authors have approved the final version of the manuscript and agree to be accountable for all aspects of the study. All persons designated as authors qualify for authorship, and all those who qualify for authorship are listed.

## Data availability

All data generated or analyzed during this study are included in this published article.
